# N-terminal Pro-Brain Natriuretic Peptide And Atrial Fibrillation

**Published:** 2009-01-07

**Authors:** Jayachandran Thejus, Johnson Francis

**Affiliations:** Calicut Medical College, Kerala, India

**Keywords:** NT-Pro-BNP, BNP, pro-BNP, atrial fibrillation, cardioversion

## Abstract

NT-proBNP is produced from both atria and ventricles. The primary regulation of production is at the synthesis level. The plasma half-life of NT-proBNP is 60-120 min. Cutoff value of NT-proBNP for diagnosis of heart failure is 125 pg/ml in the age group below 75 years and 450 pg/ml in the age group above 75 years. It increases in atrial fibrillation and drops after successful cardioversion. High levels predict development of atrial fibrillation (AF) in healthy persons with sinus rhythm (SR). Some studies concluded that baseline level predicts maintenance of SR after cardioversion of AF while some others found that it did not. Many studies have proven that it is useful in monitoring rhythm stability after cardioversion of AF. Since it is increased in many other conditions, out of which some may also cause AF, care must be taken before ascribing changes in its level to AF alone.

## Introduction

Atrial fibrillation (AF) is the most common sustained arrhythmia. There are two modalities of treatment for AF- rate control and rhythm control. In cases where it is expected that sinus rhythm (SR) will be maintained after cardioversion, rhythm control is preferred. There are many predictors of SR after cardioversion of AF, of which N-terminal pro-BNP (NT-proBNP) is a recently described one. It is presently being studied extensively in the hope that it will aid in deciding whether to go for rate control or rhythm control in AF.

## NT-proBNP

BNP is a polypeptide encoded by a gene on chromosome 1. Pro-BNP is the 108 amino-acid precursor molecule which is cleaved, by the enzyme furin, into C- and N-terminal fragments. The C-terminal fragment is the 32 amino-acid, biologically active BNP while the N-terminal fragment is the 76 amino-acid, biologically inactive NT-proBNP.

 BNP and NT-proBNP are produced in both the atria and the ventricles. These are produced not only in cardiac myocytes, but also in other cardiac cell types, especially cardiac fibroblasts. They have only a small granule storage pool. The main stimulus for their synthesis and release is wall stress, which produces a rapid gene transcription response. The primary regulation of production is at the synthesis level, not at the secretion level. At rest, 60% of secreted BNP is from the ventricles while the rest is from the atria (logically, same should apply to NT-proBNP also). The predominant source of circulating BNP (and hence, probably of NT-proBNP also) may be ventricles or atria depending on the etiology and severity of the cardiac disorder. In AF, the main source is the atria. In heart failure, the main source is traditionally thought to be the ventricles, though recent studies have found that the atria also contribute significantly, with one study concluding that atrial production is more than ventricular production [1].

The plasma half-life of BNP is 21 min while that of NT-proBNP is 60-120 min. Thus, BNP fluctuates more in response to acute hemodynamic changes while NT-proBNP is more stable over time.

The normal value of NT-proBNP depends heavily on age, with levels increasing with increasing age. The level is slightly higher in females. The FDA approved cutoff value for heart failure diagnosis is 125 pg/ml if age is <75 years while it is 450 pg/ml if age is >75 years. Some studies have defined the upper limit of normal as 340 pg/ml. Renal failure causes mild elevation of NT-proBNP levels.

NT-proBNP is usually used for the diagnosis of heart failure, especially to distinguish heart failure from respiratory disease in patients presenting with dyspnoea, and for monitoring the progress (or response to treatment) of heart failure. NT-proBNP testing is commercially available and is very expensive.

## NT-proBNP is increased in AF

 Various studies have proved that NT-proBNP is increased in AF [2-5]. The mean level in various studies is in the range of 800 to 1100 pg/ml. The main sources are probably the atria, as they have been proven to be the main sources of BNP in this setting [6,7]. The proposed mechanisms are high frequency of atrial myocyte contraction and local atrial inflammation [2].

 In AF, NT-proBNP level does not correlate either with the duration of AF or with left atrial dimensions [4]. How quickly NT-proBNP increases after the onset of AF has not been studied. It has been proven that BNP increases within 4 hours of the onset of AF; whether this applies to NT-proBNP also is not clear [8].

 NT-proBNP levels drop after successful cardioversion of AF. Danicek et al [3] found that the median value dropped from 970 pg/ml to 471 pg/ml at 24 hrs after cardioversion (but the authors did not comment on whether this achieved statistical significance or not). Shin et al [4] found that even at 11 days after cardioversion, NT-proBNP was higher in patients who were maintaining SR, compared to healthy controls, possibly due to atrial stunning and undetected paroxysmal AF.

## NT-proBNP predicts AF

Asselberg et al [9] found that in the general population, elevated NT-proBNP levels at baseline predicted the development of AF when reassessed at 4 years. The baseline median level was 62.2 pg/ml in those who eventually developed AF compared to 35.7 pg/ml in those who did not. The difference was found to be highly significant statistically (p = 0.001). Values above the 80th percentile (97 pg/ml in women and 60 pg/ml in men) were associated with an odds ratio of 2.65 for the occurrence of AF.

## Baseline NT-proBNP level predicts maintenance of sinus rhythm after cardioversion of AF (or does it?)

 Some studies concluded that baseline NT-proBNP level predicts maintenance of SR after cardioversion of AF while some others found that it did not. Mollmann et al [2] found that baseline NT-proBNP more than 900 pg/ml significantly predicts (p<0.05) persistence of AF at 4 weeks after DC version of lone AF. This study used continuous Holter monitoring for 4 weeks. The 900 pg/ml cutoff had a sensitivity of 84.2%, a specificity of 73.5%, a positive predictive value of 64% and a negative predictive value of 89.3%. The authors concluded that NT-proBNP might offer a valuable aid in deciding for or against a rhythm control strategy. Sanna et al [10] in a study published in this issue of the journal found that baseline NT-proBNP was an independent predictor of AF recurrence at 6 months after DC version. They found that NT-proBNP level above 1707 pg/ml had a specificity of 92% and a sensitivity of 36% in predicting AF recurrence at 6 months.

Danicek et al [3] found that baseline NT-proBNP did not predict whether patients would maintain SR after cardioversion of AF. Shin et al [4] found that baseline NT-proBNP was higher (1570.5 pg/ml vs 973.6 pg/ml) in patients who eventually developed a recurrence of AF, but this difference did not achieve statistical significance (p=0.23). Similarly, Buob et al [5] found that baseline NT-proBNP was higher (2996 pg/ml vs 1647 pg/ml) in patients who eventually developed a recurrence of AF, but this difference did not achieve statistical significance (p=0.37).

## NT-proBNP is useful in monitoring rhythm stability after cardioversion of AF

 Many studies have proven that NT-proBNP is useful for this purpose. NT-proBNP remains high in patients who continue to have persistent AF. The follow up level in patients who continue to be in AF is significantly higher when compared to patients who remain in SR [2,3].

NT-proBNP does not remain high in patients with paroxysmal AF after successful cardioversion. It falls; and the level at 1 month is not significantly different when compared to patients who remain in SR [2], but is significantly lower when compared to patients who have persistent AF [3]. At 6 months, the ratio of follow up level to baseline level is significantly higher in patients who have paroxysmal AF when compared to patients who remain in SR [3].

## NT-proBNP is increased in other conditions also

 Since NT-proBNP is increased in many other conditions, out of which some may cause AF also, care must be taken before ascribing changes in its level to AF alone. These conditions include left heart failure, right heart failure due to pulmonary embolism or cor pulmonale, acute coronary syndromes, stable coronary artery disease, left ventricular hypertrophy due to hypertension or aortic stenosis, right ventricular hypertrophy due to pulmonary hypertension and renal failure. Also note that the levels are higher in the elderly, in females and in those with a low BMI.

## Summary

NT-proBNP is increased in AF. The degree of baseline elevation of NT-proBNP before cardioversion of AF has not been convincingly shown to predict long term maintenance of sinus rhythm after cardioversion. After cardioversion of AF, serial monitoring of NT-proBNP is useful in monitoring for recurrence of AF.

## References

[R1] Luchner A (1998). Differential atrial and ventricular expression of myocardial BNP during evolution of heart failure. Am Am J Physiol.

[R2] Mollmann H (2008). NT-ProBNP predicts rhythm stability after cardioversion of lone atrial fibrillation. Circ J.

[R3] Danicek V (2008). Sinus rhythm restoration after atrial fibrillation: the clinical value of N-terminal pro-BNP measurements. Pacing Clin Electrophysiol.

[R4] Shin DI (2005). Plasma levels of NT-pro-BNP in patients with atrial fibrillation before and after electrical cardioversion. 2005.

[R5] Buob A (2006). Discordant regulation of CRP and NT-proBNP plasma levels after electrical cardioversion of persistent atrial fibrillation. Pacing Clin Electrophysiol.

[R6] Inoue S (2000). Atrium as a source of brain natriuretic polypeptide in patients with atrial fibrillation. J Card Fail.

[R7] Goetze JP (2006). Atrial secretion of B-type natriuretic peptide. Eur Heart J.

[R8] Tsuchida K (2004). Influence of paroxysmal atrial fibrillation attack on brain natriuretic peptide secretion. J Cardiol.

[R9] Asselbergs FW (2008). N-terminal pro B-type natriuretic peptide levels predict newly detected atrial fibrillation in a population-based cohort. Neth Heart J.

[R10] Sanna T (2009). Baseline NT-Pro-BNP Levels and Arrhythmia Recurrence in Outpatients Undergoing Elective Cardioversion of Persistent Atrial Fibrillation: A Survival Analysis. Indian Pacing Electrophysiol J.

